# A multi-omics pipeline integrating machine learning and spatial-cellular analysis identifies SASH1 as a prognostic biomarker and therapeutic target in head and neck squamous cell carcinoma

**DOI:** 10.1097/JS9.0000000000003647

**Published:** 2025-10-16

**Authors:** Ziwei Dai, Xiaofeng Shan, Yifan Kang, Yutong Chen, Qiushi Feng, Zhigang Cai, Shang Xie

**Affiliations:** a Department of Oral and Maxillofacial Surgery, Peking University School and Hospital of Stomatology, Beijing, China; b National Center for Stomatology, Beijing, China; c National Clinical Research Center for Oral Diseases, Beijing, China

**Keywords:** head and neck squamous cell carcinoma (HNSCC), machine learning, multi-omics integration, SASH1, tumor microenvironment

## Abstract

**Background:**

Head and neck squamous cell carcinoma (HNSCC) is a highly aggressive malignancy with a poor prognosis, necessitating the discovery of novel and reliable molecular biomarkers for improved clinical management. Traditional bulk transcriptomic analyses often mask the cellular heterogeneity and spatial complexity of the tumor microenvironment, limiting the identification of robust biomarkers. This study aimed to identify and validate key driver genes in HNSCC through a comprehensive multi-omics and machine learning-based approach.

**Materials and methods:**

Transcriptomic data from multiple GEO datasets (GSE29330, GSE6631, GSE138206) and the TCGA-HNSC cohort were integrated and analyzed to identify consensus differentially expressed genes (DEGs). A suite of four machine learning algorithms (LASSO, SVM-RFE, XGBoost, Boruta) was employed to screen for core candidate genes. The cellular origins and spatial distribution of these core genes were subsequently dissected using public single-cell (GSE215403) and spatial transcriptomics (GSE252265) data. Finally, the expression of the key gene, SAM and SH3 domain-containing 1 (SASH1), was validated at the protein level via Western blot in HNSCC cell lines, and its clinical and therapeutic value was assessed through survival, clinical correlation, and drug sensitivity analyses.

**Results:**

An integrated analysis of bulk transcriptomic data identified 159 consensus DEGs, from which four core genes (COL1A1, EMP1, MYH11, SASH1) were robustly selected by all four machine learning algorithms. Multi-omics validation revealed that SASH1 was specifically downregulated within the malignant cell population and its expression was spatially exclusive from the COL1A1-high fibrotic stromal regions. Western blot confirmed the significant downregulation of SASH1 protein in HNSCC cells compared to controls. Importantly, low SASH1 expression was significantly associated with poorer overall survival in the TCGA cohort (*P* < 0.05), a prognostic value not observed for the other core genes. Functional analyses linked SASH1 to critical pathways including cell cycle and adhesion. Furthermore, SASH1 expression levels correlated with sensitivity to multiple targeted drugs, including ATR and Aurora kinase inhibitors.

**Conclusion:**

By systematically integrating multi-platform transcriptomics, machine learning, and multi-dimensional validation, this study identifies SASH1 as a robust prognostic biomarker and a potential predictor of therapeutic response in HNSCC. The established multi-omics pipeline provides a meaningful framework for biomarker discovery and highlights SASH1 as a promising target for advancing precision medicine in HNSCC.

## Introduction

Head and neck squamous cell carcinoma (HNSCC) is a prevalent malignancy originating from the epithelial tissues of the oral cavity, nasopharynx, hypopharynx, larynx, and other upper aerodigestive tract regions, accounting for over 90% of all head and neck tumors^[1]^. According to the Global Cancer Observatory 2020, HNSCC results in over 930 000 new cases and approximately 450 000 deaths annually, with rising incidence in South Asia, Southeast Asia, and certain low- and middle-income countries^[2]^. Despite advances in surgery, radiotherapy, and chemotherapy, the 5-year overall survival rate for HNSCC remains around 50%, with advanced-stage patients frequently facing challenges such as local recurrence and distant metastasis^[3]^. Current clinical practice relies heavily on histopathological evaluation and the TNM staging system for HNSCC assessment. However, these approaches have limitations in predicting treatment response and guiding personalized therapies. Consequently, there is an urgent need to identify sensitive, specific, and reproducible molecular biomarkers to enhance early diagnosis, prognosis evaluation, and individualized treatment strategies for HNSCC.

The widespread adoption of high-throughput sequencing has significantly advanced the study of tumor molecular mechanisms. Public databases, such as the Gene Expression Omnibus (GEO) and The Cancer Genome Atlas (TCGA), compile extensive transcriptomic data, enabling cross-dataset analyses to improve the robustness of research findings^[4,5]^. However, analyses based solely on bulk transcriptomics are often hampered by technical noise, platform-specific biases, and, most critically, the masking of cellular heterogeneity^[6]^. A bulk tissue sample represents an average signal from a complex mixture of malignant cells, stromal cells, and infiltrating immune cells. This averaging effect can obscure cell type-specific gene expression changes and fail to capture the intricate spatial organization of the tumor microenvironment (TME), which is crucial for understanding tumor progression and immune evasion.

Despite significant progress in HNSCC transcriptomic research, several limitations persist. First, many studies rely on single datasets, which can lead to non-reproducible findings due to sample and platform-specific biases^[7]^. Second, while some studies have employed machine learning for feature selection, the use of a single algorithm can be susceptible to model bias or overfitting, potentially failing to identify the most biologically significant molecules^[8,9]^. More importantly, even robust biomarkers identified from bulk data lack cellular and spatial resolution. It remains unclear whether a gene’s differential expression originates from the cancer cells themselves or from changes in the TME, and how its expression is organized within the tissue architecture. This knowledge gap limits our mechanistic understanding and the clinical translatability of many putative biomarkers.

To address these multifaceted limitations, this study establishes a comprehensive multi-omics pipeline to systematically identify and validate molecular biomarkers with high diagnostic and prognostic potential in HNSCC. We began by integrating transcriptomic data from multiple public databases (GEO and TCGA) and employed a suite of four complementary machine learning algorithms to robustly screen for core candidate genes. Crucially, to overcome the limitations of bulk analysis, we then leveraged single-cell and spatial transcriptomics to dissect the cellular origins and in situ topographical patterns of these core genes within the HNSCC ecosystem. Finally, the protein-level expression of the ultimate key candidate, SAM and SH3 domain-containing 1 (SASH1), was validated experimentally via Western blot. By constructing a multi-layered evidence chain spanning large-scale computational screening to high-resolution cellular, spatial, and protein-level validation, this study aims not only to identify novel, reliable biomarkers but also to provide deeper mechanistic insights into their roles. These findings hold promise for advancing precision medicine and improving clinical outcomes for patients with HNSCC. In adherence to best practices for reporting research involving computational tools, the structure and reporting of this study comply with the TITAN 2025 guidelines^[10]^.

**HIGHLIGHTS**

- A multi-omics pipeline integrating machine learning and experimental validation robustly identifies SASH1 as a key downregulated gene intrinsic to malignant HNSCC cells.
- Single-cell and spatial analyses reveal that SASH1 loss is associated with malignant progression and is spatially exclusive from the pro-tumorigenic fibrotic stroma.
- Low SASH1 expression predicts poorer patient survival and correlates with sensitivity to targeted therapies, highlighting its value as a prognostic and predictive biomarker in HNSCC.

## Materials and methods

### Data sources and preprocessing

To integrate transcriptomic data for HNSCC, this study retrieved two microarray datasets, GSE29330 (GPL570 platform) and GSE6631 (GPL8300 platform), along with an independent validation dataset, GSE138206 (GPL570 platform), from the GEO database. RNA-sequencing data from the TCGA-HNSC project were downloaded via the GDC Data Portal using the TCGAbiolinks package. All data processing was conducted in the R environment. GEO datasets were obtained using the GEOquery package, with probe annotation performed using corresponding GPL files. Custom R scripts mapped probe IDs to gene symbols, averaging expression values for genes with multiple probes to construct a gene-level expression matrix. TCGA-HNSC RNA-sequencing data were transformed into log2(FPKM + 1) format and normalized using the normalizeBetweenArrays function from the limma package to eliminate batch effects and platform differences. Expression matrices for tumor and normal tissues were extracted based on sample grouping, and design and contrast matrices were constructed. Linear models were fitted using the lmFit function, followed by Bayesian correction with the eBayes function. DEGs were identified using |log2 Fold Change (logFC)| > 1 and adjusted *P*-value (adj.*P*.Val) < 0.05. DEG expression patterns were visualized using heatmaps (pheatmap package), and fold changes with statistical significance were presented in volcano plots (ggplot2 package). Mean expression, fold change, and standard error (SE) for each DEG were calculated to support subsequent functional annotation and modeling.

### Batch effect correction and data integration

To integrate the GEO datasets GSE29330 and GSE6631, expression matrices were merged using gene names as the primary key. To address potential batch effects from different data sources, the removeBatchEffect function from the limma package (via the sva package) was applied. Batch information was extracted from original file names and included as a batch factor in the correction model to remove systematic biases due to technical differences. Boxplots and principal component analysis (PCA) scatter plots were generated before and after batch correction to visualize the correction effect. Boxplots displayed the distribution of expression values across samples, while PCA scatter plots, based on the first two principal components, illustrated sample clustering to reflect changes in batch effects.

### Differential expression analysis

To identify significant DEGs associated with HNSCC, differential expression analysis (DEA) was performed on GSE29330, GSE6631, and TCGA-HNSC datasets using the limma package in R. For GSE29330 and GSE6631, background correction and normalization were applied to construct gene expression matrices. Linear models were fitted using the lmFit function, followed by Bayesian correction with the eBayes function to compute differential expression statistics, with DEGs selected based on |log2FC| > 1 and adj.*P*.Val < 0.05. For TCGA-HNSC, raw HTSeq-count data were normalized using the TMM method and transformed with the voom function to meet linear modeling assumptions, followed by the same limma pipeline and criteria (|log2FC| > 1, adj.*P*.Val < 0.05). The expression matrices of GSE29330 and GSE6631 were merged, normalized using the normalizeBetweenArrays function, and subjected to the same DEA pipeline to identify DEGs. The intersection of DEGs from individual GSE29330, GSE6631, merged dataset, and TCGA-HNSC analyses yielded 159 consensus DEGs for subsequent analyses.

### Functional enrichment analysis

To investigate the biological functions of the 159 consensus DEGs, Gene Ontology (GO) and Kyoto Encyclopedia of Genes and Genomes (KEGG) pathway enrichment analyses were conducted using the clusterProfiler, org.Hs.eg.db, and enrichplot packages in R. Gene symbols were converted to Entrez IDs to generate the gene list for analysis. GO enrichment, covering biological process (BP), cellular component (CC), and molecular function (MF) categories, was performed using the enrichGO function, with significant terms selected based on *P*-value and adjusted *P*-value (*q*-value) thresholds of 0.05. Results were visualized as bubble plots showing the top 10 enriched GO terms, sorted by gene ratio. KEGG pathway enrichment was conducted using the enrichKEGG function with the human genome as the reference, applying the same *P*-value and *q*-value thresholds (0.05).

### Machine learning feature selection

To identify key feature genes from the 159 consensus DEGs, four machine learning feature selection methods were employed: LASSO Regression (Least Absolute Shrinkage and Selection Operator), Support Vector Machine Recursive Feature Elimination (SVM-RFE), XGBoost, and Boruta. LASSO Regression applies L1 regularization to shrink feature coefficients, selecting features by setting coefficients of unimportant features to zero. The optimal penalty parameter *λ* was determined via cross-validation to identify genes most predictive of disease. SVM-RFE recursively eliminates features with the lowest contribution to classification performance, ranking features by weight and retaining those most critical to model accuracy. XGBoost gradient boosting tree algorithm evaluates feature importance based on metrics like gain and coverage during tree construction, optimizing the loss function through iterations to rank disease-relevant genes. Based on random forests, Boruta algorithm compares the importance of real features against randomly generated “shadow” features, retaining genes significantly outperforming shadow features through iterative comparisons. The number of genes and their importance scores were recorded for each method. To ensure reliability and stability, the intersection of genes selected by all four methods was identified as the final key feature genes.

### Independent dataset validation

To validate the expression differences of core genes, GSE138206 was used as the validation dataset. Four core genes were selected, and their expression differences between control and tumor groups were compared using *t*-tests. The normalized expression matrix was processed with the limma package, and core gene expression data were extracted. *T*-tests assessed expression differences, with *P*-values < 0.05 indicating significance. Results were visualized using boxplots.

### Single-cell RNA-seq data processing and analysis

To dissect the expression heterogeneity of the core genes at cellular resolution, a public HNSCC single-cell RNA sequencing (scRNA-seq) dataset (GSE215403) was downloaded from the GEO database. Data processing was primarily conducted using the Seurat R package. Following stringent quality control based on the number of detected genes, UMIs, and mitochondrial gene percentage, the data underwent normalization, identification of highly variable features, dimensionality reduction (PCA and UMAP), and unsupervised clustering. Cell clusters were then meticulously annotated manually based on canonical marker genes to identify malignant cells, fibroblasts, and various immune cell subpopulations. The expression patterns of the four core genes across different cell types were visualized using DotPlot, FeaturePlot, and VlnPlot functions. To further explore the dynamic changes within the malignant cell population, pseudotime trajectory analysis was performed on the malignant cell subset using the Monocle3 package. A cellular developmental trajectory was constructed using the learn_graph and order_cells functions, with the trajectory root being set to the malignant cell group exhibiting high SASH1 expression. Finally, the expression states of the four core genes or the cells’ vertex groups on the graph were mapped onto the trajectory for visualization using the plot_cells function.

### Spatial transcriptomics data analysis

To investigate the in situ spatial distribution of the core genes, an HNSCC spatial transcriptomics dataset generated with the 10x Genomics Visium platform (GSE252265) was obtained. The data were also processed using the Seurat package. After quality control and normalization via SCTransform, dimensionality reduction (PCA) and unsupervised clustering were performed to identify distinct spatial domains. Subsequently, these spatial domains were manually annotated into functional regions, based on the expression of key marker genes. Finally, the spatial expression patterns of the four core genes on the tissue section were visualized using the SpatialFeaturePlot function.

### Survival analysis and prognostic evaluation

To evaluate the prognostic role of core genes in HNSCC, survival analysis was conducted using TCGA-HNSC patient data. Patients were divided into high- and low-expression groups based on median gene expression. The survival package in R was used, with the survfit function fitting Kaplan-Meier survival curves to estimate survival probabilities. The Log-rank test (survdiff function) compared survival differences between groups, calculating chi-square and *P*-values. Results were visualized as Kaplan-Meier curves, showing survival time distributions with Log-rank *P*-values annotated (significance threshold: *P* < 0.05).

### Single-gene functional annotation

To explore the potential functions of SASH1 in HNSCC, Spearman correlation analysis was performed to identify genes significantly correlated with SASH1 expression in the TCGA-HNSC dataset. Correlation coefficients (*r*) were calculated, and genes with |*r*| > 0.5 and *P* < 0.00001 were selected for statistical significance. Using the clusterProfiler package, GO (covering BP, CC, and MF) and KEGG enrichment analyses were conducted on the correlated gene set, with significant terms filtered by adjusted *P*-value (*P*.adjust < 0.05). Results were visualized to illustrate SASH1-related gene functions and pathways, following the same methodology as above.

### Clinical correlation and drug sensitivity analysis

To investigate the clinical relevance and drug sensitivity of SASH1 expression in HNSCC, TCGA-HNSC data were analyzed for associations with clinical features (gender, age, stage, survival status) and drug response. Chi-square tests assessed associations between SASH1 expression and categorical variables (gender, survival status), while two-tailed *t*-tests compared SASH1 expression differences for continuous variables (age, stage), with a significance threshold of *P* < 0.05. Drug sensitivity analysis utilized IC_50_ data from oncoPredict databases. Pearson correlation analysis evaluated linear relationships between SASH1 expression and IC_50_ values, while Spearman correlation assessed nonlinear or monotonic relationships, with significant results filtered by *P* < 0.05. Results were visualized using correlation heatmaps for clinical and drug response associations, scatter plots for SASH1 expression versus specific drug IC_50_ values, and boxplots for SASH1 expression across clinical feature groups.

### Cell culture and western blot

To validate the expression of SASH1 at the protein level, this study employed the human immortalized oral keratinocyte cell line HOK and the human tongue squamous cell carcinoma cell line CAL-27 (ATCC, Manassas, VA, USA). Cells were routinely cultured in their respective recommended media and harvested upon reaching the logarithmic growth phase. Total protein was extracted using RIPA lysis buffer and quantified via the BCA assay. Equal amounts of protein were subjected to SDS-PAGE separation and subsequently transferred onto PVDF membranes. The membranes were incubated overnight at 4°C with primary antibodies against SASH1 (1:1000) and β-actin (1:5000). The following day, membranes were incubated for 1 hour at room temperature with corresponding HRP-conjugated secondary antibodies, followed by visualization using an enhanced chemiluminescence (ECL) detection system. Band intensities were scanned and semi-quantitatively analyzed using ImageJ software. All experiments were independently repeated three times to ensure reproducibility and reliability of the results.

### Statistical analysis

Statistical analyses were performed to assess the significance of results and ensure the robustness of findings. All statistical tests were conducted using R software (version 4.3). For differential expression analysis, *P*-values were adjusted for multiple comparisons using the Benjamini-Hochberg method to control the false discovery rate (FDR). A significance threshold of adjusted *P*-value (adj.*P*.Val) < 0.05 was used to identify DEGs. For survival analysis, the Kaplan-Meier method was used to estimate survival curves, with the Log-rank test applied to compare survival differences between groups. A *P*-value < 0.05 was considered statistically significant in all survival analyses. To evaluate correlations, Spearman’s or Pearson’s correlation coefficients were calculated depending on whether the data followed a linear or monotonic relationship. Correlation analysis results were visualized using heatmaps, scatter plots, and correlation plots. Statistical significance for all tests was defined as *P* < 0.05 unless otherwise stated. Statistical analyses were carried out using R packages such as limma, survminer, and corrr.

## Results

### Differential expression analysis and batch effect correction of GSE29330 and GSE6631 datasets

**Figure 1.**
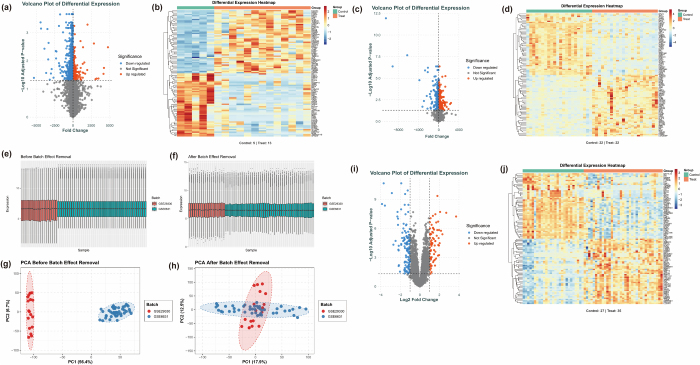
Differential gene expression and batch effect analysis in HNSCC. (a, b) Volcano plot and heatmap showing DEGs in the GSE29330 dataset. (c, d) Volcano plot and heatmap of DEGs identified in the GSE6631 dataset. (e, f) Boxplots depicting gene expression distributions before (e) and after (f) batch effect correction. (g, h) PCA plots illustrating sample clustering before (g) and after (h) removal of batch effects. (i, j) Volcano plot and heatmap of DEGs derived from the batch-corrected combined dataset.

DEA of the GSE29330 dataset identified 1599 DEGs, including 1224 downregulated and 375 upregulated genes (Fig. 1(a,b)). Analysis of the GSE6631 dataset revealed 873 DEGs, comprising 384 downregulated and 489 upregulated genes (Fig. 1(c,d)). Due to potential batch effects arising from different data sources, batch effect correction and data integration were performed on GSE29330 and GSE6631 to ensure reliability and consistency in subsequent analyses. Boxplots of sample expression distributions before and after batch correction are shown in Figure 1(e,f), where notable differences in expression distributions between the two datasets were observed before correction, and alignment was achieved post-correction. PCA further validated the correction, showing clear separation of samples before correction and a well-mixed distribution after correction (Fig. 1(g,h)). Using the batch-corrected dataset, DEA was repeated, identifying 190 DEGs (118 downregulated, 72 upregulated) (Fig. 1(i,j)), laying the foundation for subsequent functional enrichment analyses and biological interpretation.

### The GO and KEGG analyses for overlapped genes

**Figure 2.**
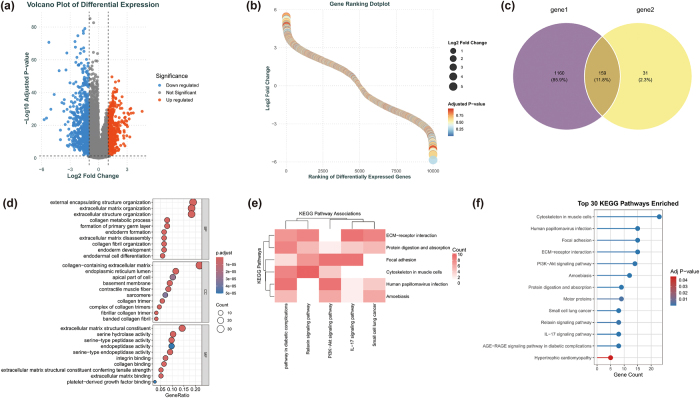
Differential expression and functional enrichment analysis of DEGs in HNSCC. (a) Volcano plot of DEGs in TCGA-HNSC dataset. (b) Gene ranking dotplot based on adjusted *P*-value. (c) Venn diagram showing overlap between TCGA and batch-corrected DEGs. (d) GO enrichment analysis of overlapped DEGs. (e) KEGG pathway correlation heatmap. (f) Top 30 enriched KEGG pathways ranked by adjusted *P*-value.

To explore the biological functions of DEGs associated with HNSCC, 1319 DEGs (549 upregulated, 770 downregulated) were identified from the TCGA-HNSC dataset, as visualized in the volcano plot (Fig. 2(a)) and gene ranking dotplot based on adjusted *P*-value (Fig. 2(b)). These were intersected with 190 DEGs from the batch-corrected dataset, yielding 159 overlapped DEGs, as shown in Figure 2(c). GO analysis of these 159 DEGs revealed significant enrichment in BP related to ECM remodeling, including ECM organization, collagen metabolic processes, and external encapsulating structure organization. For CC, DEGs were primarily associated with collagen-containing ECM, sarcomeres, and collagen trimers. MF were enriched in ECM structural constituents, serine-type endopeptidase activity, and integrin binding, suggesting key roles in ECM stability and intercellular signaling (Fig. 2(d)).KEGG analysis identified significant enrichment in pathways linked to tumorigenesis, including ECM-receptor interaction, focal adhesion, PI3K-Akt signaling, and cytoskeletal regulation in muscle cells. Additionally, the AGE-RAGE signaling pathway and IL-17 signaling pathway showed significant enrichment, indicating potential links to inflammation and metabolic dysregulation (Fig. 2(e,f)). The KEGG pathway correlation heatmap further revealed functional overlap and synergy between ECM-related pathways, PI3K-Akt, and Relaxin signaling pathways (Fig. 2(e)). Collectively, these 159 overlapped DEGs likely play critical roles in HNSCC progression by regulating ECM dynamics, cell adhesion, and key signaling pathways.

### Multi-machine learning algorithm integration and independent validation of key core genes

**Figure 3.**
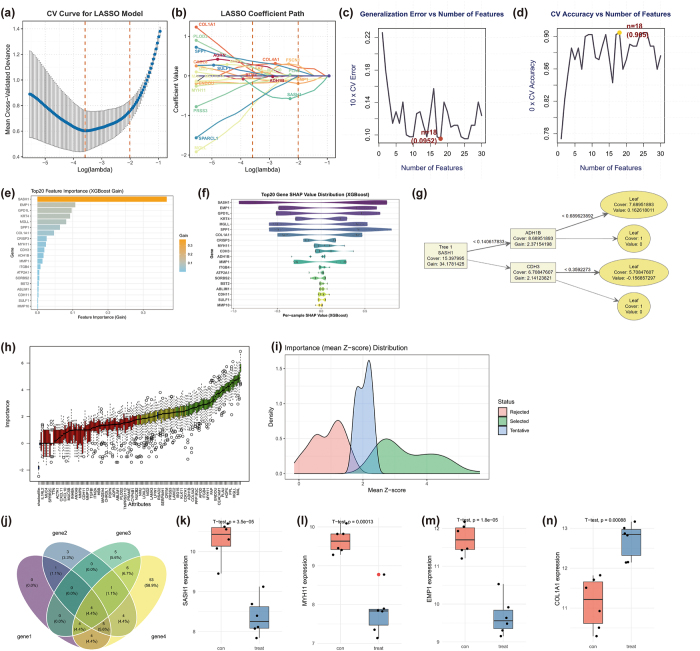
Identification and validation of key core genes using multi-ML approaches. (a, b) LASSO regression results: cross-validation curve (a) and coefficient path (b). (c, d) SVM-RFE performance: generalization error trend (c) and cross-validation accuracy/error (d). (e–g) XGBoost feature importance: top genes by gain (e), feature impact across samples (f), and decision tree structure (g). (h, i) Boruta gene classification: Z-score-based selection (h) and density distribution of selected genes (i). (j) Venn diagram showing intersection of key features from all four methods, identifying four core genes. (k–n) Independent validation of four core genes ((k) SASH1;(l) MYH11;(m) EMP1;(n) COL1A1) in GSE138206 dataset, showing significant differential expression (*P* < 0.001 for all).

To identify key predictive features from the 159 DEGs associated with HNSCC, four feature selection methods—LASSO Regression, SVM-RFE, XGBoost, and Boruta—were systematically applied, leveraging linear modeling, support vector learning, ensemble tree models, and random forest stability selection, respectively. LASSO Regression used ten-fold cross-validation on the standardized 159 DEGs to determine the optimal regularization parameter lambda (Fig. 3(a,b)). The cross-validation curve with the minimum mean squared error (MSE) marked by an orange dashed line indicates the optimal balance between bias and variance (Fig. 3(a)). The coefficient path shows gene coefficients trending toward zero with increasing lambda, with 18 genes retaining non-zero coefficients at the optimal lambda, suggesting strong predictive potential (Fig. 3(b)). SVM-RFE assessed model performance across varying feature counts (Fig. 3(c,d)). Decreasing generalization error as feature numbers increase is shown in Figure 3(c), and peak CV accuracy (CV Accuracy = 0.905) and minimum error (CV Error = 0.0952) at 18 features validate the superiority of this feature subset (Fig. 3(d)). XGBoost, based on gradient boosting, analyzed the 159 DEGs (Fig. 3(e–g)). SASH1 was highlighted as the top feature by gain, followed by EMP1, GPD1L, KRT4, and MGLL (Fig. 3(e)). SASH1’s broad impact across samples, with EMP1, GPD1L, and KRT4 also contributing consistently, is shown in Figure 3(f). The decision tree structure confirms SASH1 as the root node (Gain = 34.18) driving the initial split, followed by ADH1B and CDH3, yielding 20 high-gain genes (Fig. 3(g)). Boruta, using random forests and shadow features, categorized genes into Selected (green), Tentative (blue), and Rejected (red) based on Z-scores (Fig. 3(h,i)). The density distribution of Selected genes with significantly higher Z-scores resulted in 81 statistically significant features (Fig. 3(i)). The intersection of features from all methods (LASSO: 18, SVM-RFE: 18, XGBoost: 20, Boruta: 81) identified four core genes consistently selected: COL1A1, EMP1, MYH11, and SASH1 (Fig. 3(j)). To validate their robustness and generalizability, single-gene expression analysis was conducted on the independent validation dataset GSE138206 (excluding training datasets GSE6631 and GSE29330). Boxplots show significant expression differences for SASH1 (*P* < 0.001), MYH11 (*P* < 0.001), EMP1 (*P* < 0.001), and COL1A1 (*P* < 0.001) (Fig. 3(k–n)).

### Cellular heterogeneity of core genes in the HNSCC microenvironment via single-cell analysis

**Figure 4.**
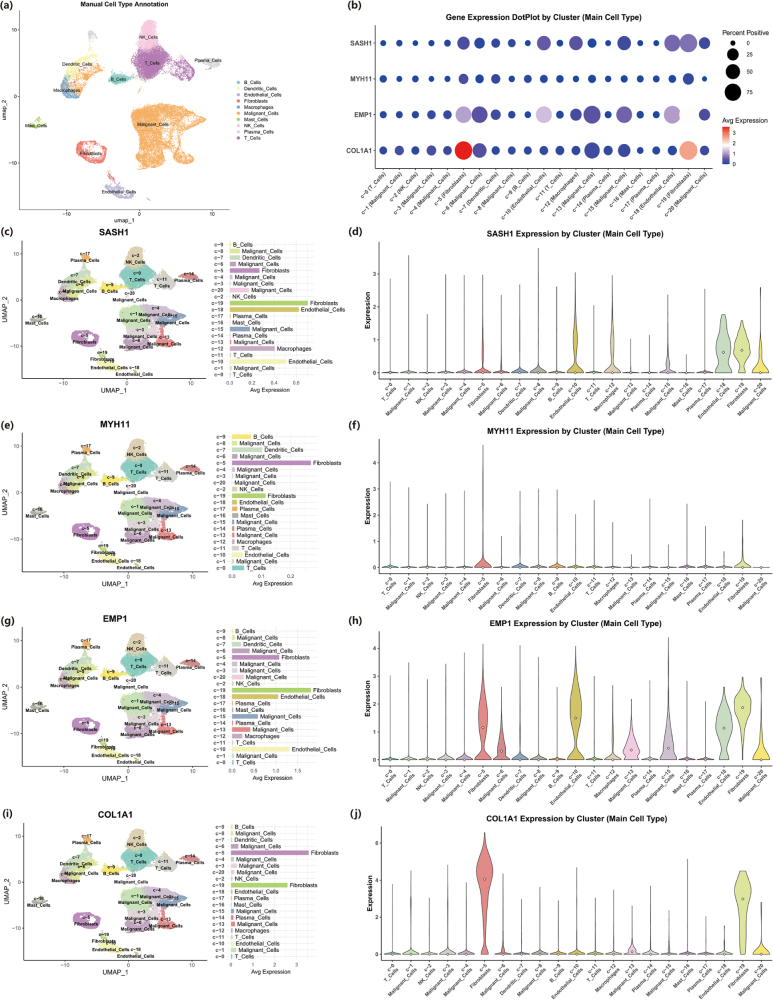
Single-cell sequencing reveals the expression profiles of SASH1, MYH11, EMP1, and COL1A1 across different cell subpopulations. (a) UMAP plot showing manual annotation of Malignant cells, Fibroblasts, Endothelial cells, and various immune cell populations. . (b) Dot plot illustrating the average expression levels and percentage of expression for SASH1, MYH11, EMP1, and COL1A1 in various cell types. (c-d) Feature plot (c) and violin plot (d) of SASH1 expression on the UMAP, showing its relatively low expression in malignant cells but higher expression in stromal (Fibroblasts, Endothelial) and immune (B cells, Plasma cells) populations. (e-f) Feature plot (e) and violin plot (f) of MYH11 expression, indicating minimal baseline expression across nearly all cell types. (g-h) Feature plot (g) and violin plot (h) of EMP1 expression, demonstrating its expression in multiple cell types, with notable presence in Fibroblasts and specific subpopulations of Malignant cells. (i-j) Feature plot (i) and violin plot (j) of COL1A1 expression, highlighting its highly specific and strong expression almost exclusively in Fibroblasts.

**Figure 5.**
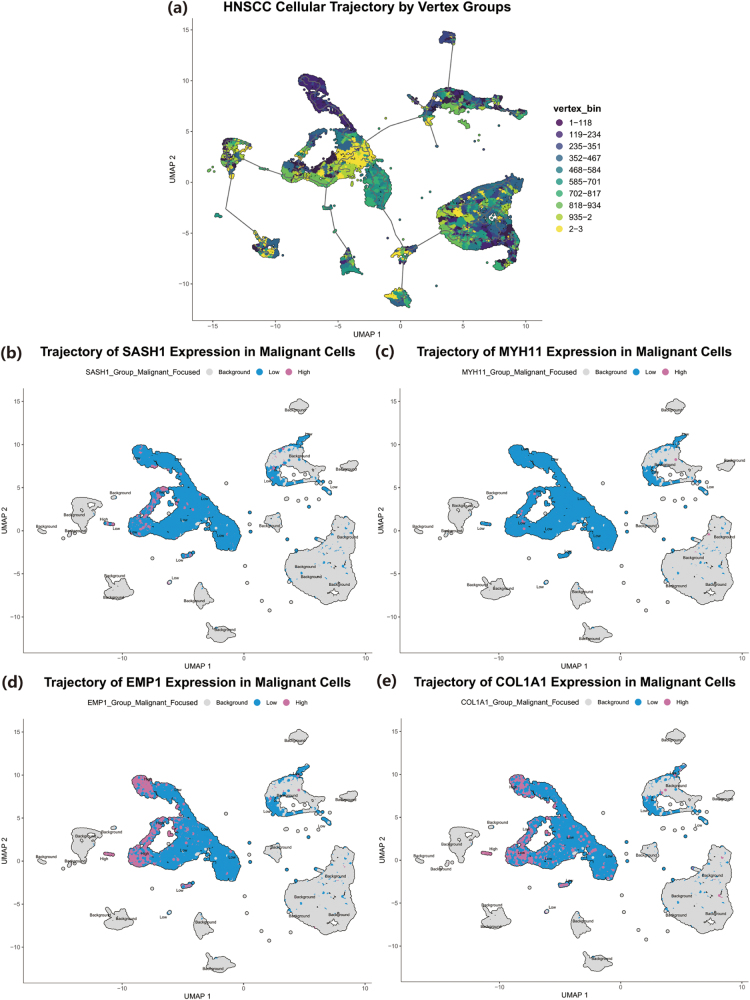
Pseudotime trajectory analysis of malignant cells. (a)UMAP plot of the cellular differentiation trajectory for malignant cells in HNSCC, with colors representing the pseudotime progression. (b) Expression pattern of SASH1 along the malignant cell trajectory, showing the vast majority of malignant cells maintain a low-expression state (blue). (c) Expression pattern of MYH11 along the malignant cell trajectory. (d) Expression pattern of EMP1 along the malignant cell trajectory. (e) Expression pattern of COL1A1 along the malignant cell trajectory. In (b-e), cells are colored based on expression levels: low (blue) and high (pink), with non-malignant cells shown as a gray background.

To resolve the expression profiles of the four core genes (COL1A1, EMP1, MYH11, SASH1) at single-cell resolution, public HNSCC single-cell transcriptomic data were analyzed. Through unsupervised clustering and subsequent manual annotation, a total of 11 major cell types were identified, including malignant cells, fibroblasts, endothelial cells, as well as various immune cells such as T cells, B cells, plasma cells, macrophages, NK cells, dendritic cells, and mast cells. These cells collectively form the cellular atlas of the tumor microenvironment (Fig. 4(a)). A comprehensive analysis of the expression levels and percentages of the four core genes across all cell types revealed distinct patterns (Fig. 4(b)). COL1A1 expression was highly concentrated in the fibroblast clusters (c-6, c-19), where it showed the highest average expression and a positive rate of over 75%. EMP1 exhibited a broader distribution, with moderate expression levels detected in fibroblasts (c-6, c-19), some malignant cell clusters (c-8), and endothelial cells (c-10, c-18). MYH11 demonstrated extremely low average expression and a low percentage of positive cells across all identified cell types. In contrast to the others, SASH1 expression was primarily observed in stromal and immune cells, with higher average expression in fibroblasts, endothelial cells, B cells, and plasma cells. Conversely, its average expression level was relatively low in multiple subpopulations annotated as malignant cells (e.g., c-1, c-3, c-4, c-6, c-8, c-13, c-15, c-20). To further illustrate the expression distribution of each gene, feature plots and violin plots were generated (Fig. 4(c-j)). The feature plot for SASH1 intuitively showed that its expression signals (warm colors) were mainly distributed in non-malignant cell regions, while appearing as a low-expression “cold zone” in the large malignant cell areas (Fig. 4(c)). The corresponding violin plot confirmed that the median expression of SASH1 was close to zero in all malignant cell clusters (Fig. 4(d)). MYH11 maintained a minimal baseline expression across all cell types (Fig. 4(e,f)). EMP1 expression was detected in multiple clusters, with its peaks observed in c-6 (Fibroblasts) and c-8 (Malignant Cells) (Fig. 4(g,h)). The expression pattern of COL1A1 was the most specific, with its high expression signals almost exclusively restricted to the two fibroblast clusters, c-6 and c-19, and being near-zero in all other cell types (Fig. 4(i,j). Furthermore, to investigate the expression dynamics of these core genes within the malignant cell population, a cellular trajectory was constructed (Fig. 5(a)). After stratifying malignant cells into high and low expression groups based on the median expression within this population, visualization revealed different dynamic patterns for the four genes. For SASH1 and MYH11, nearly all malignant cells were classified into the low-expression state (blue), with only a few cells showing high expression (Fig. 5(b,c)). In contrast, EMP1 and COL1A1 displayed evident expression heterogeneity within the malignant cell population, with both high-expressing (pink) and low-expressing (blue) subpopulations present. Notably, the EMP1-high cells clustered on a specific branch in the upper-left portion of the trajectory plot (Fig. 5(d)), while the COL1A1-high cells also showed a clustered distribution, primarily located in two small branches on the left and upper-right of the trajectory (Fig. 5(e)).

### Spatial topography of core genes in HNSCC tissues

**Figure 6.**
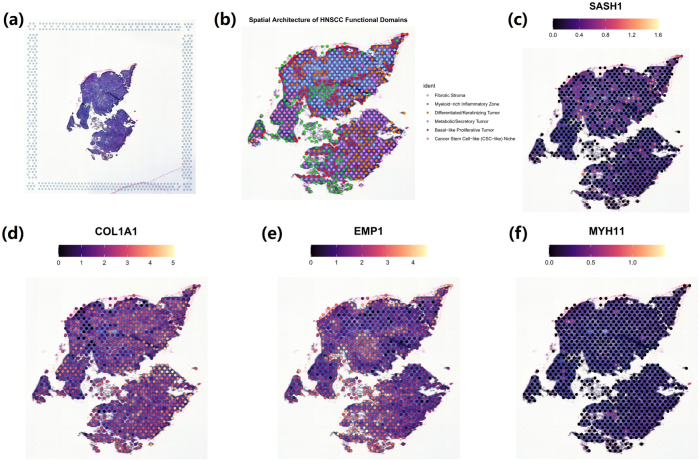
Spatial transcriptomics analysis of the tissue distribution of SASH1, COL1A1, EMP1, and MYH11. (a) Hematoxylin and Eosin (H&E) stained image of the tissue section. (b) Cell type annotation plot from spatial transcriptomics data, illustrating the cellular composition of different tumor regions. (c) Spatial expression map of SASH1, demonstrating its enrichment primarily within the Myeloid-rich Inflammatory Zone and Fibrotic Stroma, while showing low expression in the core tumor domains. (d) Spatial expression map of COL1A1, highlighting its highly specific localization to the Fibrotic Stroma. (e) Spatial expression map of EMP1, showing a broader distribution with notable expression in the stromal regions and at the tumor-stroma interface. (f) Spatial expression map of MYH11, confirming its low and diffuse expression throughout the entire tissue section.

To investigate the spatial localization of the four core genes within the in situ tissue environment, spatial transcriptomics was performed on a representative HNSCC sample. The H&E staining of this sample illustrates its histopathological morphology, showing dense tumor cell nests and surrounding stromal structures (Fig. 6(a)). Following unsupervised clustering of the spatial transcriptomic profiles, the resulting spatial domains were manually annotated into six functionally distinct partitions based on the expression patterns of key marker genes. These partitions clearly delineated the functional heterogeneity within the tissue, primarily including a Fibrotic Stroma zone, a Myeloid-rich Inflammatory Zone, and four distinct tumor subregions: Differentiated/Keratinizing Tumor, Metabolic/Secretory Tumor, Basal-like Proliferative Tumor, and a Cancer Stem Cell-like (CSC-like) Niche (Fig. 6(b)). Upon this annotated spatial atlas, the expression of the four core genes was visualized, revealing starkly different and highly structured spatial distribution patterns. SASH1 expression was notably low across the vast majority of the tissue, particularly within the large, dense areas annotated as Fibrotic Stroma and most tumor subregions. Its expression signals were almost uniformly cool-colored (low expression) across the entire tissue section, indicating its general downregulation in the established tumor ecosystem (Fig. 6(c)).

In striking contrast to SASH1, the expression of COL1A1 clearly delineated the stromal architecture of the tumor. Its high expression signals (warm colors) were almost exclusively and intensely enriched in the regions annotated as Fibrotic Stroma. Conversely, its expression was minimal in all tumor subregions and the myeloid inflammatory zone. This result provides a vivid spatial demonstration of the fibrotic microenvironment and highlights a clear pattern of spatial exclusion between the COL1A1-high fibrotic stroma and the SASH1-low tumor areas (Fig. 6(d)). The other two core genes also displayed unique spatial niches. High expression signals for EMP1 were prominently localized at the tumor-stroma interface, particularly concentrated in the Differentiated/Keratinizing Tumor and Metabolic/Secretory Tumor regions that directly abut the Fibrotic Stroma. This pattern suggests EMP1 may play a role in the interaction between epithelial tumor components and the surrounding fibrotic matrix (Fig. 6(e)). The expression of MYH11 was generally low and more diffuse across the tissue. While its signal was slightly more detectable in parts of the Fibrotic Stroma compared to the tumor core, it did not show the strong, specific enrichment pattern observed for the other genes (Fig. 6(f). In summary, these results provide a detailed depiction of the fine-grained spatial organization of the four core genes within the HNSCC tumor microenvironment.

### Expression differences and prognostic value of key genes in HNSCC

**Figure 7.**
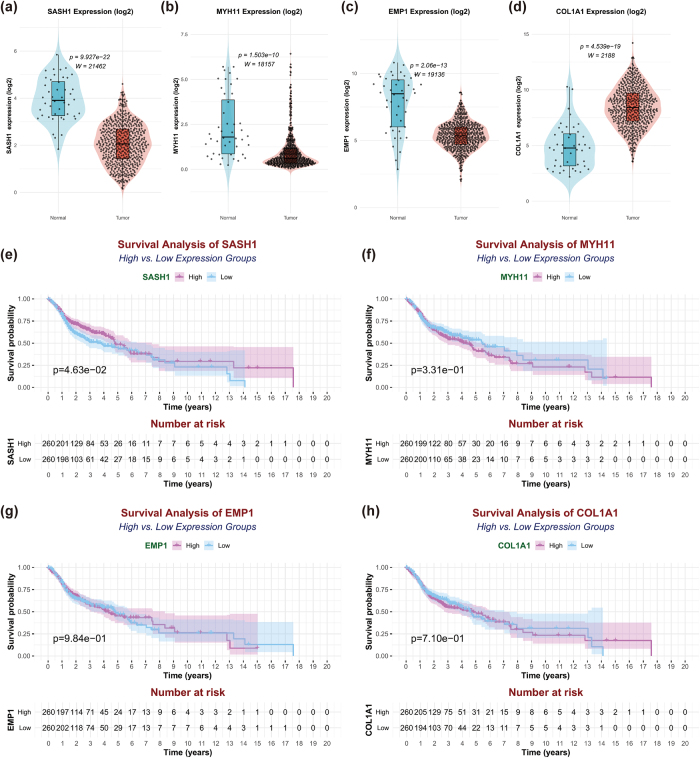
Expression profiles and overall survival analysis of four core genes in TCGA-HNSC dataset. (a–d) Violin plots showing the log2-transformed expression levels of SASH1, MYH11, EMP1, and COL1A1 in tumor and normal tissues from the TCGA dataset. The statistical significance of differences between groups was evaluated using the Wilcoxon rank-sum test. (e–h) Kaplan-Meier survival curves comparing overall survival rates between high and low expression groups for each gene (SASH1, EMP1, MYH11, and COL1A1). Patients were divided into two groups based on median expression levels of the respective genes. The log-rank test was used to assess the statistical significance of survival differences between these groups.

To further investigate the expression differences and biological significance of the four key genes (COL1A1, EMP1, MYH11, and SASH1) in HNSCC, single-gene expression analysis was performed using the TCGA-HNSC cohort (Fig. 7(a-d)). Significant expression differences between tumor and normal tissues were revealed for all four genes (*P* < 0.05). COL1A1 and EMP1 were significantly upregulated in tumor tissues, while MYH11 and SASH1 were significantly downregulated. Patients were stratified into high- and low-expression groups based on median gene expression, and Kaplan-Meier survival curves were generated (Fig. 7(e-h)). SASH1 expression showed a trend toward association with prognosis (*P* = 0.0463), with the low-expression group exhibiting lower survival probability, suggesting SASH1 as a potential favorable prognostic marker, though further validation is needed. In contrast, COL1A1 (*P* = 0.710), MYH11 (*P* = 0.331), and EMP1 (*P* = 0.984) showed no statistically significant survival differences between high- and low-expression groups, indicating that their expression may not directly impact overall survival or may require larger sample sizes for further validation.

### Functional annotation and pathway enrichment analysis of SASH1-related genes

**Figure 8.**
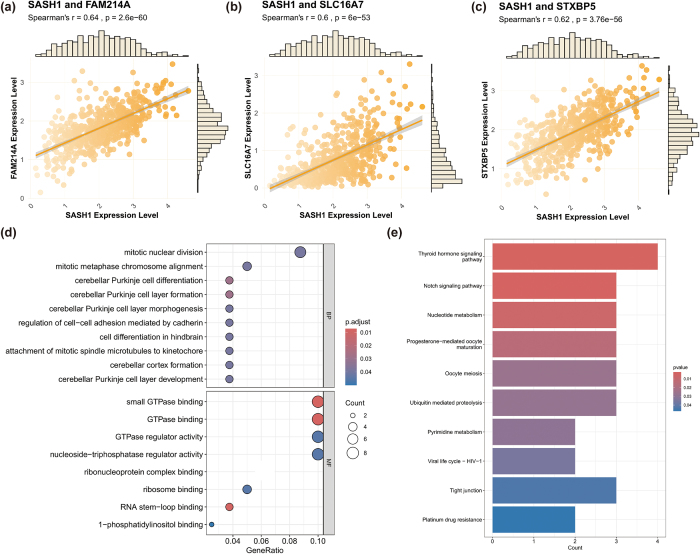
Correlation and functional enrichment analysis of SASH1 in HNSCC. (a–c) Significant positive correlations between SASH1 and FAM214A, SLC16A7, and STXBP5. (d) GO enrichment analysis showing biological processes and molecular functions. (e) KEGG pathway enrichment analysis highlighting key signaling and metabolic pathways.

To explore the biological functions of SASH1, Spearman correlation analysis identified genes significantly correlated with SASH1 expression (Fig. 8(a-c)). Strong positive correlations were observed with FAM214A (*r* = 0.64, *P* = 2.6e−60), SLC16A7 (*r* = 0.60, *P* = 6e−53), and STXBP5 (*r* = 0.62, *P* = 3.76e−56), suggesting functional synergy. GO and KEGG enrichment analyses were performed on this correlated gene set to investigate BP and MF, and enriched pathways (Fig. 8(d,e)). Significant enrichment in processes such as mitotic nuclear division, cerebellar Purkinje cell differentiation, and cell adhesion regulation was revealed, as well as molecular functions like small GTPase binding and ribonucleoprotein complex binding (Fig. 8(d)). Enrichment in pathways including thyroid hormone signaling, Notch signaling, nucleotide metabolism, progesterone-mediated oocyte maturation, oocyte meiosis, ubiquitin-mediated proteolysis, and tight junctions was identified (Fig. 8(e)). These pathways are critical in cell division, differentiation, metabolic regulation, and signal transduction, supporting the hypothesis that SASH1 plays a significant role in regulating diverse cellular functions.

### Association analysis of SASH expression with clinical features and drug sensitivity

**Figure 9.**
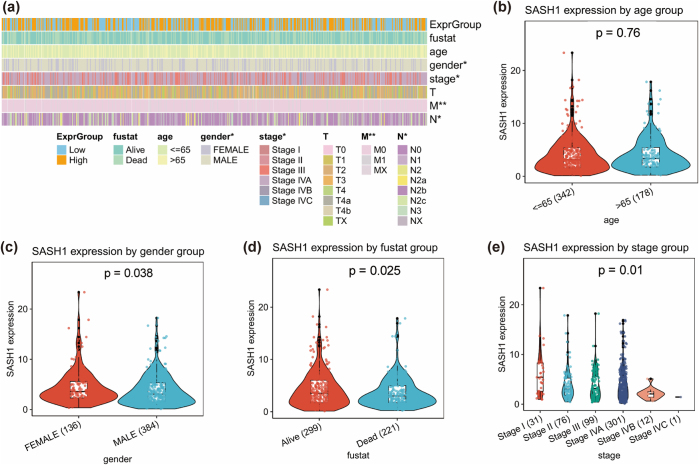
Clinical associations of SASH1 expression in HNSCC. (a) Heatmap displaying SASH1 expression across clinical subgroups including expression group, age, gender, stage, and survival status (fustat). (b-e) Box plots illustrating SASH1 expression levels by age group (b), gender group (c), survival status group (d), and stage group (e), with *P*-values indicating statistical significance. The overall statistical significance among all stage groups was determined using the Kruskal-Wallis test, yielding a p-value of 0.01.

**Figure 10.**
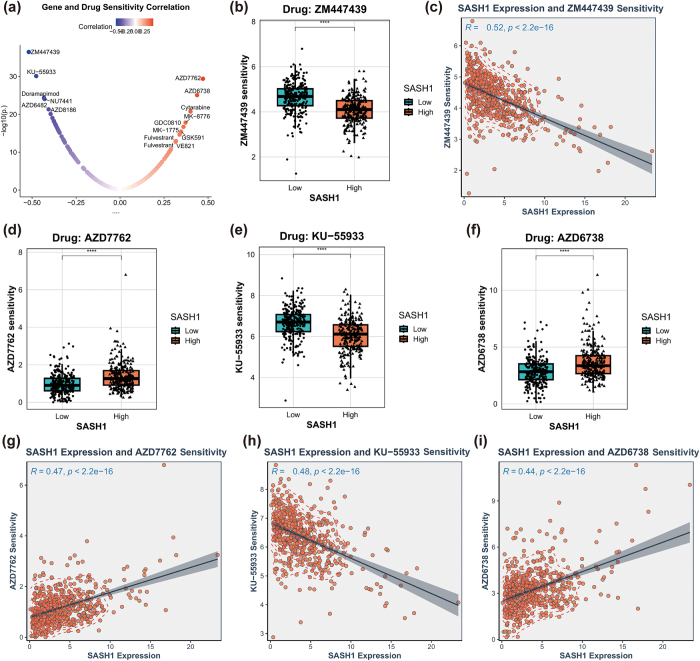
Correlation analysis of SASH1 expression with drug sensitivity in HNSCC. (a) Volcano plot illustrating the correlation between gene expression and drug sensitivity, with significant drugs (ZM447439, KU-55 933, AZD7762, AZD6738) highlighted. (b, d, e, f) Box plots comparing drug sensitivity (ZM447439, AZD7762, KU-55 933, AZD6738) between low and high SASH1 expression groups. (c, g, h, i) Scatter plots showing the correlation between SASH1 expression and sensitivity to ZM447439 (c), AZD7762 (e), KU-55 933 (g), and AZD6738 (i), with *R*^2^ and *P*-values indicated.

To assess the clinical relevance of SASH1, its expression was analyzed for associations with clinical features (Fig. 9(a-e)). Heatmaps and boxplots showed no significant expression differences across age groups (*P* = 0.76), indicating no association with age. However, SASH1 expression differences were statistically significant for survival status (*P* = 0.025), gender (*P* = 0.038), and clinical stage (*P* = 0.01), suggesting associations with prognosis, gender differences, and tumor progression. Drug sensitivity analysis revealed significant correlations between SASH1 expression and drug response (Fig. 10(a-i)). A volcano plot identified ZM447439 as having the strongest negative correlation with SASH1 expression (*r* ≈ −0.5, *P* < 1e−30) (Fig. 10(a)). Boxplots and scatter plots confirmed that high SASH1 expression was associated with reduced sensitivity to ZM447439 (*R* = −0.52, *P* < 2.2e−16) (Fig. 10(b-c)). Similarly, KU-55 933 also exhibited a negative correlation with SASH1 expression (*R* = −0.48, *P* < 2.2e−16) (Fig. 10(e, h)), further supporting the potential role of SASH1 in drug resistance. Conversely, AZD7762 and AZD6738 showed positive correlations with SASH1 expression, indicating enhanced drug sensitivity in samples with high SASH1 expression (*R* = 0.47 and *R* = 0.44, respectively, *P* < 2.2e−16) (Fig. 10(d, g, f, i)). These findings highlight the potential value of SASH1 in precision medicine, as it may influence the sensitivity to various drugs, thereby guiding personalized treatment strategies.

### Western blot validation confirms SASH1 downregulation at the protein level

**Figure 11.**
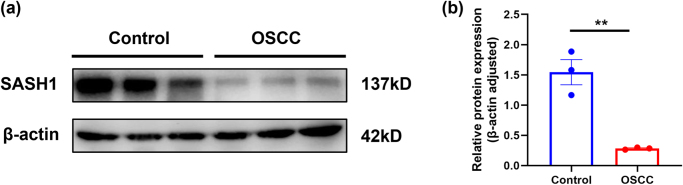
The protein expression of SASH1 is downregulated in OSCC. (a) The expression level of SASH1 protein in Control and OSCC was detected by Western blot. Β-actin was used as an internal loading control. (b) Quantitative analysis of the Western blot results. Data are presented as mean ± SD. **P < 0.01.

To validate the expression changes of SASH1 in HNSCC at the protein level, a Western blot analysis was performed. The results showed a significant change in the SASH1 protein expression level in the OSCC group compared to the Control group. Representative Western blot bands clearly demonstrated that a distinct SASH1 protein band at approximately 137 kDa was detected in the Control group cells, whereas the intensity of this band was markedly reduced in the OSCC group cells. The expression of the internal control, β-actin (~42 kDa), remained stable between the two groups, indicating equal protein loading (Fig. 11(a)). To quantify this result, densitometric analysis of the bands from three independent experiments was performed. The relative protein expression level, after normalization to β-actin, revealed that the SASH1 protein level in the OSCC group was significantly lower than that in the Control group (Fig. 11(b), *P* < 0.01).

## Discussion

HNSCC is one of the most common malignant tumors in the head and neck region, characterized by a high mortality rate and invasive progression^[11]^. Although treatment options, including surgery, radiotherapy, and chemotherapy, have improved in recent years, patients with advanced stages of the disease often face challenges such as local recurrence and distant metastasis, resulting in a 5-year survival rate that remains low, around 50%^[12]^. Traditional diagnostic methods, such as histopathological examination and TNM staging, are widely used in clinical practice; however, their effectiveness in predicting treatment response and guiding personalized therapy is limited. There is an urgent need to identify new molecular biomarkers to improve early diagnosis, enhance the reliability of prognostic evaluation, and aid in the optimization of individualized treatment strategies^[13]^.

While traditional bulk transcriptomic analysis is a powerful tool for screening differentially expressed genes, its “averaged” readouts obscure the complex cellular heterogeneity within tumors and lead to the loss of critical spatial-organizational information. This has limited the in-depth mechanistic investigation and clinical translation of many potential biomarkers. To systematically overcome these limitations, this study constructed an integrated analytical framework, moving from macro-level screening to multi-dimensional, in-depth validation. We first robustly screened a four-gene core signature, consisting of SASH1, COL1A1, EMP1, and MYH11, from a vast pool of genes by integrating multi-platform bulk data and applying a machine learning model suite comprising four complementary algorithms. The innovation of this study lies in the progressive, in-depth deconstruction of this machine learning-derived gene signature. We employed single-cell sequencing to delve into the cellular level, which not only resolved the cellular origins of the gene signature but, more critically, pinpointed the downregulation of SASH1 as an intrinsic event within malignant cells. Subsequently, by using spatial transcriptomics, we provided spatial evidence at the in-situ tissue level, revealing a pattern of spatial exclusion between SASH1 expression and the fibrotic tumor microenvironment. Finally, western blot experiments validated the low expression of SASH1 at the protein level. Through an interlocking chain of validation that progresses from computational discovery to cellular deconstruction, spatial localization, and ultimately protein verification, we systematically confirmed the significant value of SASH1 as a core biomarker in HNSCC.

In the post-genomic era, high-throughput sequencing technologies have generated massive volumes of transcriptomic data, offering unprecedented opportunities to understand the complexity of tumors at a systems level. However, a core challenge in bioinformatics is to accurately identify the “critical few” driver genes with clinical translational potential from tens of thousands of candidates^[14]^. While traditional differential expression analysis can screen numerous candidates, it often struggles to distinguish core driver genes from secondary effect molecules. It is against this backdrop that machine learning algorithms have emerged as powerful filters for biomarker discovery, owing to their robust pattern recognition and high-dimensional data processing capabilities^[15]^. Capitalizing on this advantage, our study established a multi-algorithm, cross-validated machine learning screening framework. Instead of relying on a single algorithm, which could introduce bias, we systematically integrated four models with distinct principles: LASSO, which uses L1 regularization for feature sparsification^[16]^; SVM-RFE, which iteratively eliminates features^[17]^; XGBoost, based on ensemble learning^[14]^; and Boruta, which employs random forests and “shadow” features for stable selection^[18]^. This approach ensures that only genes demonstrating strong predictive power across multiple distinct models are ultimately identified as core candidates. The results showed that SASH1 was not only one of the core genes unanimously selected by all four algorithms, but its feature importance score, as measured by the “Gain” value in the XGBoost model, also ranked highest among all candidate genes. This quantitative result indicates that within the constructed classification model, the expression level of SASH1 is one of the most important predictors for distinguishing between HNSCC and normal tissue samples. Therefore, the machine learning pipeline in this study played a critical screening role, effectively pinpointing four high-priority research leads, including SASH1, from 159 consensus differentially expressed genes. This provides a data-driven and targeted basis for subsequent single-cell, spatial transcriptomic, and experimental validations.

From the perspective of cell-intrinsic mechanisms, the loss of SASH1 expression is a key factor driving the malignant progression of HNSCC. Our single-cell trajectory analysis revealed a progressive downregulation of SASH1 expression during the evolution of malignant cells, suggesting that SASH1 plays a vital role in suppressing malignant phenotypes. To investigate its molecular mechanism, we performed functional enrichment analysis on SASH1’s co-expression network. The results showed that its co-expressed genes were significantly enriched in several critical biological processes, including “mitotic nuclear division,” “cell adhesion,” “ubiquitin-mediated proteolysis,” and the “Notch signaling pathway.” Based on these findings, we propose a hypothesis: SASH1 may exert its tumor-suppressive function by coordinately regulating multiple signaling pathways. First, it may act as a negative regulator of the cell cycle by inhibiting the “mitotic nuclear division” process, thereby restricting abnormal cell proliferation^[19]^. Second, in maintaining tissue architecture, SASH1 positively regulates “cell adhesion”^[20]^, including cell-cell junctions and cell-matrix adhesion^[21]^, to preserve epithelial integrity and thus limit cancer cell invasion and metastasis. This mechanism is consistent with reports in other cancers where it inhibits invasion by regulating the FAK signaling pathway^[22]^. Furthermore, SASH1 may be involved in the “ubiquitin-mediated proteolysis” pathway^[23]^, playing a role in maintaining cellular protein homeostasis to ensure the proper degradation of key proteins, thereby sustaining normal cell differentiation and function. Particularly important is the association of SASH1 with the “Notch signaling pathway.” Given the context-dependent dual role of Notch signaling in HNSCC^[24,25]^, the loss of SASH1 expression could lead to an imbalance in the Notch pathway, causing its aberrant activation and consequently promoting processes related to tumorigenesis and development, such as cell self-renewal, proliferation, and survival^[26]^. In summary, the loss of SASH1 may collectively promote the malignant transformation of HNSCC cells by disrupting multiple interconnected cellular programs.

Another significant finding of this study reveals the potential extrinsic function of SASH1 at the level of the tumor microenvironment, suggesting it may act as a key molecule regulating tumor-stroma interactions. Our spatial transcriptomic analysis uncovered a histological feature with significant spatial heterogeneity: a clear spatial exclusion between SASH1-low malignant cell regions and COL1A1-high fibrotic stromal regions. This observation supports an innovative hypothesis: the loss of SASH1 expression is not only a marker of intrinsic cancer cell malignancy but may also be a signaling event that initiates the remodeling of the tumor microenvironment. Specifically, SASH1-deficient cancer cells may, through paracrine mechanisms such as secreting cytokines like TGF-β, activate adjacent fibroblasts, inducing their transformation into pro-tumorigenic cancer-associated fibroblasts (CAFs)^[27,28]^. These activated CAFs interact with cancer cells through complex signaling networks. On one hand, they extensively synthesize and deposit extracellular matrix components, represented by COL1A1, to construct a fibrotic microenvironment that promotes tumor invasion and metastasis^[29,30]^. On the other hand, they further enhance cancer cell stemness, epithelial-mesenchymal transition (EMT), invasive capabilities, and therapeutic resistance^[31]^. Therefore, SASH1 may be a critical molecular node whose expression status links the intrinsic malignant transformation of tumor cells with the pro-tumorigenic remodeling of the extrinsic microenvironment. The level of SASH1 not only affects the cell’s own adhesion properties^[20]^ but also modulates its interaction with the surrounding microenvironment, thereby playing a central role in the progression of HNSCC.

Beyond its biological functions revealed at the cellular and tissue levels, the value of SASH1 as a core biomarker in HNSCC is ultimately solidified by its strong association with clinical outcomes. A pivotal finding in our multi-gene prognostic analysis was that, among the four multi-dimensionally validated core genes, only the low expression of SASH1 showed a statistically significant correlation with poorer overall survival in patients (*P* < 0.05), whereas COL1A1, EMP1, and MYH11 did not exhibit independent prognostic value. This discovery not only provides decisive evidence from a clinical cohort supporting the tumor-suppressive role of SASH1 but also highlights its immense potential as an independent prognostic biomarker for HNSCC. This is further corroborated by our clinical correlation analysis, where SASH1 expression levels were significantly correlated with survival status, gender, and clinical stage, indicating that its loss is closely linked to tumor progression and malignancy^[32]^. More significantly, this study uncovers the potential value of SASH1 in guiding personalized therapy for HNSCC. Our drug sensitivity analysis revealed that SASH1 expression levels are significantly correlated with the efficacy of multiple targeted drugs. Specifically, high SASH1 expression was negatively correlated with sensitivity to the Aurora kinase inhibitor (ZM447439) and the ATM kinase inhibitor (KU-55 933), possibly because SASH1-mediated stability of cell cycle or DNA damage repair pathways reduces the tumor cells’ dependency on these specific kinases^[33]^. Conversely, high SASH1 expression was positively correlated with sensitivity to the Chk1/2 inhibitor (AZD7762) and the ATR inhibitor (AZD6738). The underlying mechanism may be that functional SASH1 enhances checkpoint responses to DNA replication stress, thereby amplifying the cytotoxic effects of these DDR pathway inhibitors^[34,35,36]^. These findings have substantial clinical translational potential, suggesting that SASH1 expression could serve as a predictive biomarker: for patients with high SASH1 expression, combining ATR/Chk inhibitors may be an effective sensitization strategy, whereas for those with low SASH1 expression, potentially ineffective Aurora kinase/ATM inhibitors should be considered for avoidance. Particularly given that ATR inhibitors (e.g., AZD6738) can enhance the radiosensitivity of HNSCC cells^[36]^, exploring the combination of these inhibitors with standard chemoradiotherapy in SASH1-high patients, especially in p53-deficient tumors, represents a highly promising direction for overcoming therapeutic resistance^[37]^.

In summary, the core strength of this study lies in the construction and execution of an integrated research framework that extends from macroscopic computational screening to microscopic, multi-dimensional validation. By combining multi-algorithm machine learning with single-cell and spatial transcriptomics, as well as protein-level experiments, we have not only robustly identified SASH1 as a important biomarker for HNSCC but also systematically elucidated its intrinsic mechanism as a tumor suppressor and its extrinsic potential in regulating the tumor microenvironment. This has been achieved from multiple perspectives, including its cellular origin, spatial localization, and dynamic evolution, thereby providing a solid biological foundation for its clinical translation. However, this study also has certain limitations. First, our bioinformatics analysis was primarily based on public databases. Although the sample size was large, data heterogeneity and potential batch effects could still have influenced the results. Second, while our in vitro experiments confirmed the differential expression of SASH1 at the cell line level, its function requires further validation in more complex in vivo models, such as organoids. Furthermore, the precise molecular mechanisms by which SASH1 regulates the Notch pathway or interacts with fibroblasts await deeper experimental exploration. Looking ahead, this study opens up several valuable directions for future work. First, gain-of-function and loss-of-function experiments should be conducted on SASH1 in HNSCC cells and animal models using CRISPR/Cas9 gene-editing technology to directly verify its effects on tumor proliferation, invasion, and matrix remodeling. Second, developing specific antibodies against SASH1 and performing large-scale immunohistochemistry (IHC) staining on clinical samples would allow for the establishment of a standardized SASH1 protein expression scoring system. This would be crucial for validating its clinical utility as an independent prognostic marker in prospective cohorts, potentially paving new avenues for personalized precision medicine in HNSCC.

## Data Availability

The datasets used in this study are publicly available from the following repositories. The microarray datasets (GSE29330, GSE6631), independent validation dataset (GSE138206), single-cell RNA-sequencing dataset (GSE215403), and spatial transcriptomics dataset (GSE252265) were retrieved from the GEO database (https://www.ncbi.nlm.nih.gov/geo/). The RNA-sequencing data and corresponding clinical information for the HNSCC cohort were obtained from TCGA database via the GDC Data Portal (https://portal.gdc.cancer.gov/). All data can be accessed freely and used for research purposes.

## Research registration unique identifying number (UIN)

This study is a secondary data analysis of publicly available datasets and did not recruit new human participants; therefore, registration in a clinical trials database is not applicable.
